# Comprehensive Assessment of the Effect of Multi-Cropping on Agroecosystems

**DOI:** 10.3390/plants13101372

**Published:** 2024-05-15

**Authors:** Jovita Balandaitė, Kęstutis Romaneckas, Rasa Kimbirauskienė, Aušra Sinkevičienė

**Affiliations:** Agronomy Faculty, Agriculture Academy, Vytautas Magnus University, Studentu Str. 11, 53361 Akademija, Kaunas District, Lithuania; kestutis.romaneckas@vdu.lt (K.R.); rasa.kimbirauskiene@vdu.lt (R.K.); ausra.sinkeviciene@vdu.lt (A.S.)

**Keywords:** maize, hemp, faba bean, multifunctional crop, complex analysis, CEI

## Abstract

Multi-cropping is becoming an increasingly popular technique in agriculture to tackle major and complex agroecosystem problems such as biodiversity and soil fertility loss, erosion and degradation, increased greenhouse gas emissions, etc. Comprehensively assessing the impact of multi-cropping intensity on agroecosystems is a new and still under-researched approach that can provide a better understanding of the impact of individual indicators on the overall functioning of biodiverse agroecosystems. Data from a stationary field experiment using multi-cropping at the Vytautas Magnus University Experimental Station between 2020 and 2022 were used to carry out this study. The study included maize, hemp, and faba bean as single, binary, and ternary crops. A complex assessment approach (CEI value) was used to determine the impact of these crops on the agroecosystem, the interrelationships between the main indicators, and the strength of their effects. It was found that the ternary maize–hemp–faba bean crop had the most positive effect on the agroecosystem. The effectiveness of other crops was 2 to 35% less. The lowest value was calculated for the maize–faba bean crop.

## 1. Introduction

Intensive farming is still popular worldwide and in Lithuania, but it requires large investments without considering the problem of environmental pollution to maximize yields and profits [1]. Multi-cropping allows for the development of sustainable agriculture, in which high yields can be achieved with less investment in terms of money and time, and, with the right choice of crops, on less fertile soils. For example, 70–90% of faba beans in Latin America are grown as intercrops with maize or potato [2].

Multi-cropping can involve several agricultural crops that are grown in the same field but differ in their biological and agrotechnical characteristics, as well as the length of their growing season [3]. Studies have shown that mixed cropping systems have a higher yield potential than individually grown crops [4]. Maize is a major source of food for humans and livestock in many developing countries [5]. As the climate warms, maize will be increasingly grown in Lithuania to produce not only grain but also green biomass [6]. Hemp is mostly grown for its fiber or seeds, which are rich in starch, protein, and oil [7]. Hemp produces a sufficiently high biomass and can therefore be used for energy purposes [8]. Faba beans are a source of protein; thus, their nutritional and feed value is of particular importance [9]. One of the most important functions of these crops is to fix atmospheric nitrogen, which helps maintain soil fertility. Faba beans can adapt to a wide range of climatic conditions, as shown by their worldwide distribution [10].

Most multi-crops are annuals, which have clear advantages over perennials; for example, annual crops, due to their short growing season, enable farmers to produce more goods to meet market demand [11]. In addition to their main production, these crops also produce by-products, such as harvesting residues that can be used for energy purposes or in animal husbandry. Growing multiple crops in the same field at the same time can lead to more sustainable agricultural systems, making it possible to increase biodiversity; reduce damage from diseases, pests, and weeds; reduce the need for mineral fertilizers; prevent the effects of waterlogging; and maintain the fertility and quality of the soil [12,13]. Purpose-grown multi-cropping systems (agroecosystems) offer the potential to increase organic carbon, nutrient content, soil bioactivity, etc. [14]. Multi-cropping is a valuable tool for nutrient management in crop rotations, as nutrients, such as nitrogen, taken up by one plant can be utilized by other plants in the system. It has been shown that more root biomass is produced by multi-cropping compared to single-cropping. This is due to interspecific synergy between plants [15]. Multi-cropping also stabilizes and restores soil fertility by reducing the leaching of nutrients into deeper soil layers and protects the soil from wind and water erosion [14]. Multi-cropping affects the whole soil biota by increasing the abundance, diversity, and activity of soil microorganisms [16]. When multiple crops are grown, plants compete with weeds and use solar energy more efficiently [17,18]. Regardless of all the positive aspects of multi-cropping, when following the principles of sustainable agriculture, it is necessary to gradually move to non-arable farming systems and thus increase environmental protection [19].

In our study, in a multi-functional maize–hemp–faba bean agroecosystem, the role of maize is to produce abundant biomass, the role of hemp is to produce biomass and protect against diseases and pests, and the role of faba bean is to provide ecological services. The question arises as to which agroecosystem—single, binary, or ternary—is the most efficient.

The aim of this study was to perform a comprehensive assessment in order to reveal the impact of crop diversification on agroecosystems and the interrelationships between the indicators assessed and to ascertain which indices have the greatest impact on the agroecosystem and at what level of the agroecosystem.

## 2. Results and Discussion

**Figure 1:**
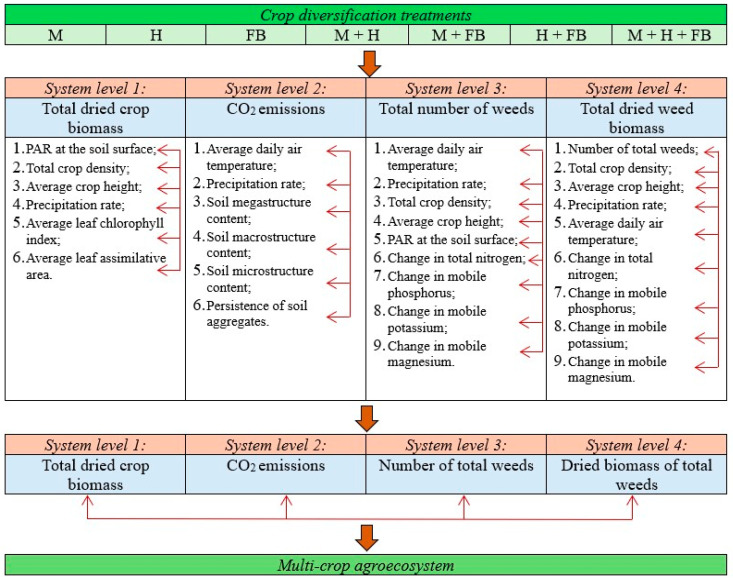
A model for comprehensive assessment of the impact of multi-cropping on agroecosystems (according to [21]).

To produce as much crop biomass per area unit as possible with minimal damage to nature, many authors recommend multi-cropping. To achieve maximum results, it is important to take advantage of the strengths of each crop grown in a multifunctional crop. This involves selecting the appropriate combinations, placement, and proportions of crop species [20]. A comprehensive multi-crop agroecosystem evaluation model is presented in Figure 1.

### 2.1. Level 1: Total Dried Crop Biomass

**Figure 2:**
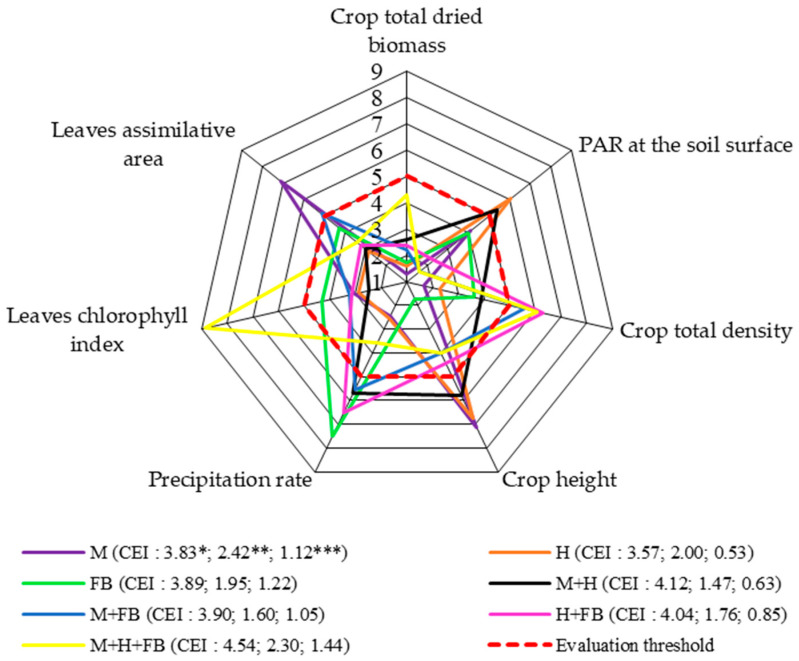
Comprehensive assessment of crop diversification in terms of total dried biomass (system level 1), 2020–2022. M: single maize crop; H: single hemp crop; FB: single faba bean crop; M + H: binary maize and hemp crop; M + FB: binary maize and faba bean crop; H + FB: binary hemp and faba bean crop; M + H + FB: ternary maize, hemp, and faba bean crop. CEI, complex evaluation index, * average of evaluation points (EPs), ** standard deviation of EPs, *** standard deviation of average of evaluation points below evaluation threshold.

The total dried crop biomass (system level 1) scores did not reach the assessment threshold. The highest score was obtained for the ternary crop of maize, hemp, and faba bean. There was also a marked advantage in terms of dried biomass scores for binary crops over single crops (Figure 2). The results show that the dried biomass of faba bean decreased at higher temperatures (r = −0.47; *p* > 0.05) because faba bean is a moderate-climate crop [22]. Tahir et al. [23] indicated that growing faba bean as an intercrop increases the biomass productivity of the crop and improves yield quality. Cai et al. [24] noted that faba bean increases the concentration of nutrients at the soil surface, leading to increased productivity of multi-crops. In their experiment, when maize was grown with rice and vetch, a significant increase in soil organic carbon was found, which was 4.12% higher than when maize was grown with rice (*p* < 0.05).

Crop productivity in any type of cropping system relies primarily on the interception of photosynthetically active radiation (PAR) at the crop canopy and the conversion of intercepted radiation into biomass, known as radiation use efficiency (RUE) [25]. Photosynthetically active radiation scores at the soil surface were found to be above the assessment threshold for hemp in a single crop and in a binary crop with maize. The assessment scores for single-cropped maize and faba bean did not reach the assessment threshold but were close to it.

The highest total crop density scores at the end of the vegetative season were obtained for the binary maize–faba bean and hemp–bean crops and the ternary crop.

The average crop height was highly affected by the cultivation of single crops, as the scores for single crops of maize and hemp were well above the assessment threshold, whereas the score for the single faba bean crop was significantly lower (Figure 2).

A correlation analysis by Litvinova et al. [26] revealed a significant effect of maize height on green biomass yield. They found that as maize height increased, the cob biomass decreased, which significantly decreased the total green biomass.

The precipitation assessment scores were below the assessment threshold for single crops of maize and hemp and the ternary crop. According to Birthal et al. [27], multi-cropping has a positive effect on the distribution of precipitation over the soil surface, and thus soil moisture is retained longer.

The scores for the crop development indicators were unevenly distributed. The leaf chlorophyll index scores were above the assessment threshold due to the influence of the ternary crop. In the case of single and binary crops, the leaf chlorophyll index scores were below the assessment threshold. The leaf assimilated area scores for the ternary and binary crops were not above the assessment threshold, except for the binary maize and faba bean crop, for which the scores were higher and above the assessment threshold.

At system level 1, the highest CEI values were found for the ternary crop, proving that the ternary crop had the greatest impact on the dried canopy biomass (Figure 2).

### 2.2. Level 2: CO_2_ Emission from the Soil

Soil respiration is a good proxy for soil biological activity, as it releases carbon dioxide (CO_2_) to the atmosphere because of the decomposition of soil organic matter [28]. Increased CO_2_ concentrations lead to increased photosynthesis and thus higher crop biomass [29].

**Figure 3:**
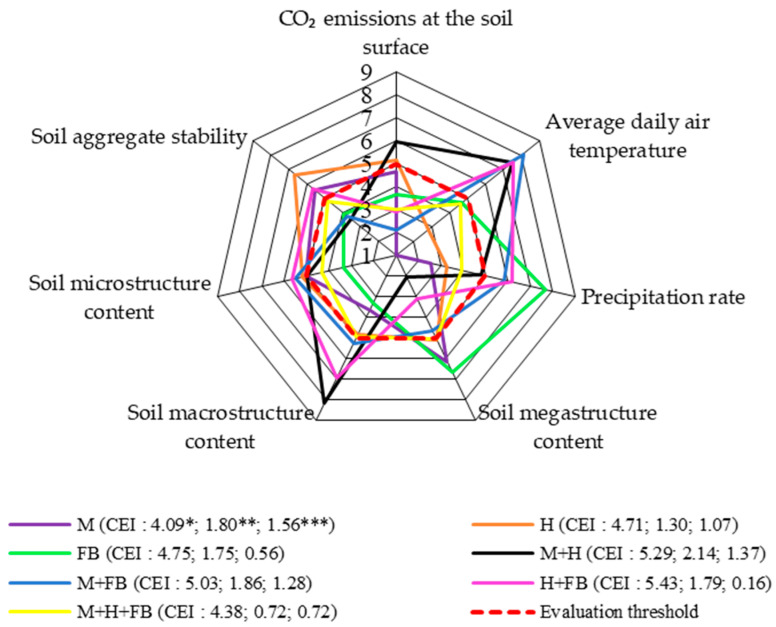
Comprehensive assessment of crop diversification in terms of CO_2_ respiration from the soil (system level 2), 2020–2022. M: single maize crop; H: single hemp crop; FB: single faba bean crop; M + H: binary maize and hemp crop; M + FB: binary maize and faba bean crop; H + FB: binary hemp and faba bean crop; M + H + FB: ternary maize, hemp, and faba bean crop. CEI, complex evaluation index, * average of evaluation points (EPs), ** standard deviation of EPs, *** standard deviation of average of evaluation points below evaluation threshold.

In our experiment, regarding the agrobiological characteristics of the soil under multi-cropping, the single hemp crop, and the binary maize–hemp crop had the greatest influence. The assessment scores for these crops were above the threshold. The CO_2_ score for the single maize crop was very close to the assessment threshold but did not exceed it (Figure 3). Hu et al. [30] found that integrating a binary maize–wheat crop with ploughless agriculture could effectively increase crop yield while reducing CO_2_ emissions from the soil and improve water use efficiency in arid areas. The scores for our tested binary crops with faba bean did not reach the assessment threshold. A study by Lupwayi et al. [31] found that, due to the ability of faba bean to fix atmospheric nitrogen, there were lower greenhouse gas emissions from the soil compared to single crops or diversified crops without faba bean.

In terms of the effect of average daily air temperature, the scores for the binary crops were above the assessment threshold. The highest precipitation effect scores were obtained for the single faba bean crop and the binary crops with faba bean (Figure 3).

The cultivation of binary crops had a greater effect on the agrophysical properties of the soil. The highest soil megastructure content scores were obtained with the single faba bean crop and the ternary crop, which were well above the assessment threshold. The soil macrostructure score was above the assessment threshold in the case of the binary maize–hemp crop. However, it should be noted that the macrostructure score for the ternary crop was also very close to the assessment threshold. Similar results were obtained by Rudinskienė, who reported that the macroaggregate content was positively affected by growing caraway in binary and ternary crops. According to the integrated assessment system, the indicators of these crops were above the assessment threshold [32,33]. In our experiment, the microstructure content scores was also influenced by the cultivation of binary crops, but these scores were only slightly above the assessment threshold, and the score for the binary crop of maize and hemp was equal to the threshold. The lowest microstructure content score was obtained with the single faba bean crop. Li et al. [34] carried out a meta-analysis of 1924 observations and reported that compared to single-cropping, multi-cropping led to increased soil macroaggregate content, while microaggregate content decreased. In our experiment, it was found that increasing the amount of megastructure in the soil decreased the amount of microstructure (r = −0.80; *p* < 0.01), while increasing the amount of macrostructure had an influence on increasing the microstructure (r = 0.46; *p* < 0.05) [22].

There was an uneven distribution of scores for soil aggregate stability. The highest soil aggregate stability scores were obtained for the single crops of maize and hemp and the binary crop of hemp–faba bean. The score for the ternary crop on the soil aggregate stability was close to, but not above, the assessment threshold. More abundant and dense crop roots improve soil stability; Hauggaard-Nielsen et al. [16] noted that interactions between crops and competition between species promote crop rooting, and the cultivation of multi-crops has a positive effect on the stability of the soil structure.

CEI calculations showed that the ternary crop was the most effective for the retardment of gas respiration.

### 2.3. Level 3: Total Number of Weeds at the End of the Vegetative Season

As well as increasing the productivity of the total crop biomass per unit area, intercropping protects the main crop from the spread of weeds, diseases, and pests as biodiversity increases. Cover crops and intercrops are fast-growing and therefore able to compete well with weeds [35,36]. It should be noted that when assessing the weediness of a crop, the reverse principle of the comprehensive assessment system applies, i.e., high CEI values take on a negative meaning and indicate higher weediness of the crop.

**Figure 4:**
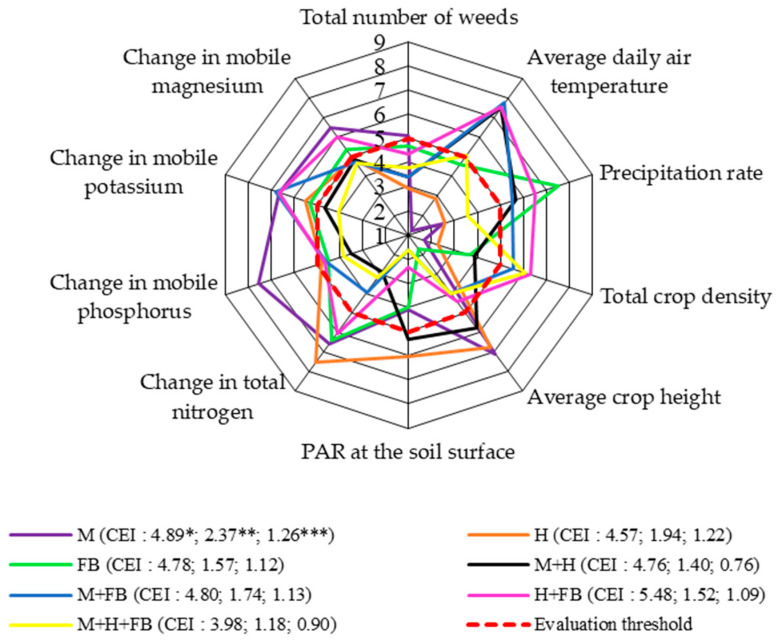
Comprehensive assessment of crop diversification in terms of total number of weeds at end of vegetative season (system level 3), 2020–2022. M: single maize crop; H: single hemp crop; FB: single faba bean crop; M + H: binary maize and hemp crop; M + FB: binary maize and faba bean crop; H + FB: binary hemp and faba bean crop; M + H + FB: ternary maize, hemp, and faba bean crop. CEI, complex evaluation index, * average of evaluation points (EPs), ** standard deviation of EPs, *** standard deviation of average of evaluation points below threshold.

In our experiment, binary and ternary crops had the greatest effect on reducing the number of weeds. The lowest weed scores were observed in the binary maize–hemp and maize–faba bean crops and the ternary crop. The total weed score was above the assessment threshold for the single maize crop and the score for the single faba bean crop was very close to the threshold (Figure 4).

The advantage of binary crops with faba bean was established by a comprehensive assessment of average daily air temperature. The assessment scores for the binary crops were significantly above the assessment threshold. In the case of the single faba bean crop, the precipitation distribution score was the highest above the threshold. Our pilot study showed that faba bean canopy height was mainly dependent on precipitation rates (r = 0.42; *p* > 0.05), as precipitation was scarce during the year of the study [22].

The highest crop density scores, which were above the assessment threshold, were obtained for the binary hemp and faba bean crop and the ternary crop. The lowest crop density score was obtained for the single maize crop. The cultivation of the single maize and hemp crops and the binary maize and hemp crop had the greatest influence on increased crop height. These crops had the highest evaluation scores, which were above the evaluation threshold. The lowest crop height assessment score was for the single faba bean crop. Livingstone et al. [37] reported that hemp sown at lower densities produced thicker and taller stems, increasing the overall green biomass of the crop. Similar results were obtained by Schafer et al. [38], showing that a denser hemp crop led to longer stems.

The scores for the assessment of radiation at the soil surface were not evenly distributed. For this indicator, single crops had an advantage over the ternary crop. The lowest radiation scores were obtained for the binary maize and hemp crop with faba bean intercropping and the ternary crop. The highest scores were obtained for single crops and the binary maize and hemp crop.

In terms of soil agrochemical properties, the scores were unevenly distributed, but there was a marked advantage of single crops over binary and ternary crops. The highest scores for total nitrogen change were found for all single crops and the binary hemp and faba bean crop. The opposite results were obtained by Rudinskienė [32], who reported that the influence of binary and ternary crops led to a significant increase in total nitrogen above the assessment threshold. The mobile phosphorus change scores were very similar for all single crops, as well as the binary and ternary crops, and the scores were very close to the assessment threshold, although they did not reach it, except for the single crop of maize, which scored well above the threshold. The scores for the change in mobile potassium for all single, binary, and ternary crops were either above or very close to the assessment threshold. Similar trends were observed for the change in mobile magnesium. Vanino et al. [39] found that crop diversification improved soil quality. In an experiment, they found increased soil organic carbon, nitrogen, and phosphorus with multi-cropping.

As in the previous level, the ternary crop had the least effect on weed number. This means that using ternary crops may be an effective method to limit the spread of weeds in agroecosystems.

### 2.4. Level 4: Total Weed Dried Biomass at the End of the Vegetative Season

**Figure 5:**
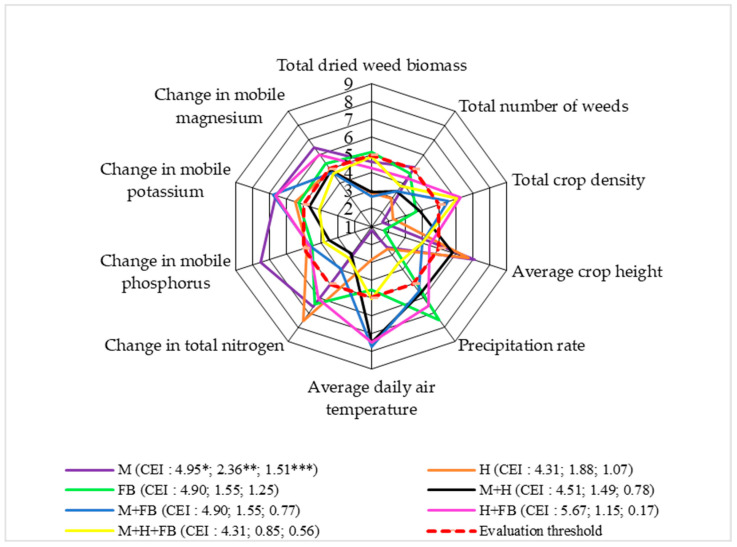
Comprehensive assessment of crop diversification in terms of total dried weed biomass at the end of the vegetative season (system level 4), 2020–2022. M: single maize crop, H: single hemp crop; FB: single faba bean crop; M + H: binary maize and hemp crop; M + FB: binary maize and faba bean crop; H + FB: binary hemp and faba bean crop; M + H + FB: ternary maize, hemp, and faba bean crop. CEI, complex evaluation index; * average of evaluation points (EPs), ** standard deviation of EPs, *** standard deviation of average of evaluation points below threshold.

The scores for weed competition showed that the binary and ternary crops had an advantage over the single crops. In the case of dried weed biomass, the evaluation scores were not above the evaluation threshold, except for the single faba bean crop. The decreased dried weed biomass was positively influenced by the cultivation of the binary maize and hemp and maize and faba bean crops and the single hemp crop, as these had the lowest evaluation scores. The cultivation of binary and ternary crops had a positive effect on reducing the weed score (Figure 5).

The crop density scores were above the assessment threshold for the binary maize and hemp crop with faba bean intercropping and the ternary crop. The cultivation of single maize and hemp crops influenced the lowest crop density scores. The average height of the crop showed the opposite results, with the highest scores for the single crops of maize and hemp. It was found that the maize grew to a lower height at higher temperatures (r = −0.45; *p* > 0.05) [22].

The precipitation scores did not reach the assessment threshold for the single maize and hemp crops and the ternary crop. Higher average daily air temperatures had a positive effect on the growth of all binary crops and the ternary crop.

When assessing the agrochemical properties of soils under multi-cropping, the total nitrogen, mobile phosphorus, potassium, and magnesium scores for single crops tended to be above the assessment thresholds and were higher compared to the scores for binary and ternary crops. Among the multi-crops, only the binary hemp and faba bean crop stood out, with scores above or close to the assessment threshold. The soil macronutrient scores for the ternary crop and the binary maize and hemp crop were below the threshold.

According to the CEI value, the ternary crop had the lowest impact on increasing weed biomass; in other words, the ternary crop stopped weed biomass growth.

### 2.5. Comprehensive Overall Assessment of All Four Levels

In the case of total crop dried biomass, the scores were unevenly distributed and did not reach the assessment threshold. The score for the ternary crop, although it did not reach assessment threshold, was close to it. The scores for the binary crops ranged from 2.22 to 2.61, and the scores were lowest for the single crops.

**Figure 6:**
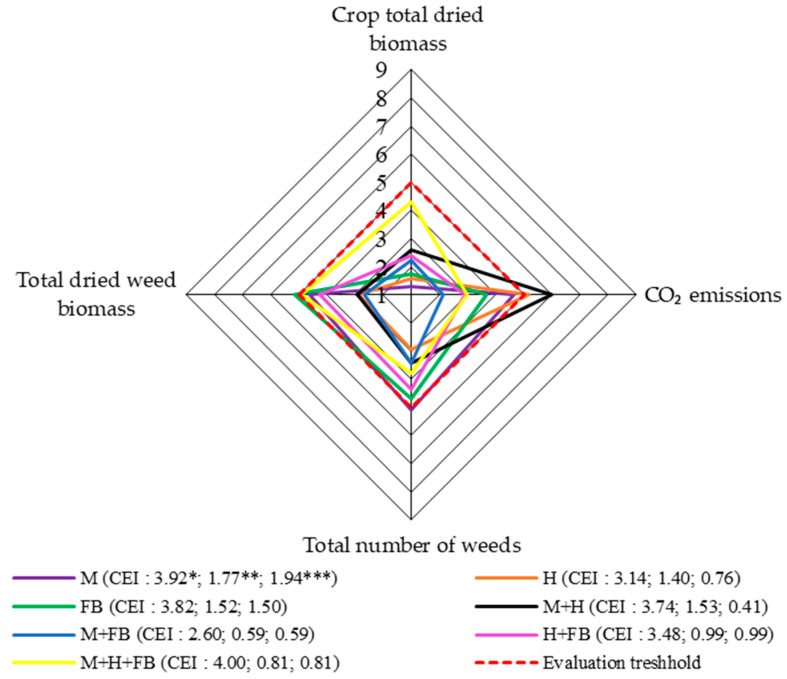
Comprehensive assessment of the impact of all four system levels on agroecosystem, 2020–2022. M: single maize crop; H: single hemp crop; FB: single faba bean crop; M + H: binary maize and hemp crop; M + FB: binary maize and faba bean crop; H + FB: binary hemp and faba bean crop; M + H + FB: ternary maize, hemp, and faba bean crop. CEI, complex evaluation index; * average of evaluation points (EPs), ** standard deviation of EPs, *** standard deviation of average of evaluation points below threshold.

The highest CO_2_ emission scores, which were above the assessment threshold, were obtained for the single crop of hemp and the binary crop of maize and hemp. The assessment score for the single maize crop was close to the threshold (Figure 6).

The most positive effects on weed numbers and dried biomass were observed with the single hemp crop and the binary maize–hemp and maize–faba bean crops. These crops had the lowest evaluation scores, which were within the evaluation threshold. In terms of the number of weeds and their dried biomass, the highest scores, which were above or close to the threshold, were obtained for the single maize and faba bean crops. Slightly lower scores, but close to the threshold, were obtained for the ternary crop and the binary hemp and faba bean crop. Thus, the cultivation of these crops had a negative effect on weediness.

The overall CEI value showed that the ternary crop had the best results or the most effective results. The study showed that assessing the impact of multi-cropping on an agroecosystem at four main levels provides a clearer understanding of the impact of the set of indicators on the overall functioning of the agroecosystem. Such an assessment framework can help decision-makers determine how best to implement multifunctional agriculture to maintain or increase yields and ecosystem service provision when considering alternative strategies for the development of new cropping systems [39]. It should be noted that the studies conducted and calculations made to assess the effect of multi-cropping were carried out under arable farming conditions. In conservation agriculture, under no-till or simplified conditions, CEI values may differ, which requires further field research.

## 3. Materials and Methods

### 3.1. Site Description

The field experiment was carried out in 2020–2022 at the Experimental Station of Vytautas Magnus University Agriculture Academy (coordinates: 54°53′7.5″ N 23°50′18.11″ E). The soil of the experimental field was a deeper gleyic saturated loam (45.6% sand, 41.7% silt, 12.7% clay) Planosol (*Endohypogleyic-Eutric Planosol-Ple-gln-w*) [40]. Soil pH_HCl_ varied from 7.3–7.8; total nitrogen content was 0.08–0.13%; humus content was 1.5–1.7%%; available phosphorus content was 189–280 mg·kg^−1^; available potassium content was 97–118 mg·kg^−1^; available sulfur content was 1.2–2.6 mg·kg^−1^; and available magnesium content was 436–790 mg·kg^−1^. The microrelief was levelled and the water regime was regulated by closed drainage.

In 2020, the average daily air temperature and precipitation during the vegetative season were close to the long-term average (since 1974). In 2021, the air temperature was high, and the distribution of precipitation was very uneven, as there was too little moisture in the middle of the vegetative season and not enough at the end. In 2022, the end of the vegetative season was unusually dry with high temperatures (Table 1).

### 3.2. Experimental Design and Agricultural Practice

There were 7 experimental treatments: single crops of maize (1), hemp (2), and faba bean (3); binary crops of maize and hemp (4), maize and faba bean (5), and hemp and faba bean (6); and a ternary crop of maize, hemp, and faba bean (7). The initial size of the plots was 8 m^2^. A total of 21 plots were investigated in the experiment. Table 2 shows the grouping of crops, the treatments, and their abbreviations.

According to the sowing plan, 50 cm row spacing was maintained when sowing a single row of maize, hemp, or faba bean. Before the experiment was set up, sown oats (*Avena sativa*) were grown on the site. After oat harvesting, straw was disked with a Väderstad Carrier 300 disk harrow (Väderstad AB, Väderstad, Sweden) and ploughed after 2 weeks with a Gamega PP-3–43 plough with semi-helical ploughshares (Gamega Ltd., Garliava, Lithuania). In the spring of each year of the experiment, when the soil reached the right moisture content, it was cultivated to a depth of 3–4 cm with a Laumetris KLG-3.6 seedbed cultivator (Laumetris Ltd., Keleriškės village, Lithuania)). Subsequently, the plots were formed and fertilized with NPK 15:15:15 (200 kg ha^−1^) in the first year and NPK 5:15:30 + 5S (200 kg ha^−1^) in the second and third years of the experiment. Maize (*Zea mays* L.) (Pioneer hybrid P8105), hemp (*Cannabis sativa* L.) (variety Austa SK), and faba bean (*Vicia faba* L.) (variety Vertigo) were sown manually in a continuous manner in the experimental plots as single, binary, and ternary crops according to a predetermined sowing scheme. After the weeds had germinated in abundance, the inter-rows were mowed twice using a hand hoe weeder. Synthetic pesticides were not used during the experiment. The agro-techniques of the experiment are well described by Balandaitė [22].

### 3.3. Methods and Analysis

#### 3.3.1. Soil Agrochemical Properties

In each experimental year after crop harvesting, 0–25 cm layer soil samples were taken for macronutrient evaluation. Laboratory analyses were conducted to determine the levels of the main macro-elements (N, P, K, and Mg) and the pH of the soil. The analyses were carried out at the Agrochemical Research Laboratory of the Lithuanian Centre for Agriculture and Forestry.

*Soil structure and stability*. Soil samples were collected with a shovel after sowing the crops before inter-row mowing from 0–25 cm soil layer. A Retsch sieving apparatus (Retsch Lab Equipment, VERDER Group, The Netherlands) and a set of sieves were used to determine the soil structure. The water stability (wet sieving) of soil aggregates was determined by the Retsch wet sieving device from a previously dry sieved soil fraction of 1–2 mm. The results of the tests are expressed as percentages [41].

*CO_2_ concentration and emissions*. These parameters were determined by infrared gas analyzer (IFGA). An LI-8100A portable soil respiration system with an 8100–103 camera was used. A 20 cm diameter ring was hammered into each plot in the spring and 3 measurements were taken: at the beginning, in the middle, and at the end of the crop vegetative season.

*Photosynthetically active radiation.* Tests were carried out during the vegetative season at the beginning of faba bean flowering. Photosynthetically active radiation (PAR) was measured with an HD 9021 RAD/PAR radiometer (FAR E m^−2^, 400–700 nm). PAR was measured in different arcs of the crop: at the ground surface, at one-half height, and above the crop (background). This indicator is expressed as a percentage of background irradiance [22].

*Crop density*. At the end of the vegetative season, crop density was evaluated by determining the productivity of multiple crops. Density was evaluated at a minimum of 5 locations in the plot, in a 0.5 m longitudinal row. An average sample was taken. The crop density is given as plants per m^2^.

*Crop development indicators.* These were determined in the second half of the vegetative season by assessing the chlorophyll index and leaf assimilative area. The leaf assimilative area (cm^2^) was determined using a Win Dias leaf area meter (Delta-T Devices Ltd., Cambridge, UK). The chlorophyll index of the leaves was measured with a CCM-200 Plus chlorophyll meter.

*Crop biometric and productivity indices.* These were measured at harvest time to assess crop height and dried biomass. A total of 36 spots were generated for the study. The biomass samples were dried in a thermostat at 105 °C to constant weight [42].

*Weed flora*. This parameter was determined by assessing the number of weeds at the end of the crop vegetative season. The number of weeds (m^−2^) and the dried biomass (g m^−2^) at the end of the vegetative season were studied. Weeds were uprooted, dried to air-dry biomass, and weighed, and a botanical analysis of species composition was performed [43].

The variations in the indices (variables) described above and the units of measurement are presented in Table 3.

#### 3.3.2. Statistical Analysis

Correlation and regression analyses of the data were carried out using the the statistical software package SYSTAT, version 10 [44]. A comprehensive evaluation of the multi-cropping effect on the agroecosystem was carried out based on the methodologies of Lohmann [45] and Heyland [46]. An example of the CEI calculations is shown in Supplementary Materials, Table S1 from reference [21].

## 4. Conclusions

A comprehensive assessment of the main indices (variables) in a multi-crop agroecosystem found that in terms of total dried crop biomass, the effects of ternary and binary crops with maize and hemp on the agroecosystem were the highest. The assessment of total weed numbers and dried weed biomass showed that the scores for indices of the binary hemp and faba bean crop were significantly above the assessment threshold, indicating it had an advantage compared to single and multi-crops. The assessment of CO_2_ emissions showed that the binary crops had the greatest impact on the agroecosystem.

The calculation of CEI showed that the ternary maize–hemp–faba bean crop had the least impact on gas emissions and increased weed abundance (levels 2–4) and the greatest effect on total dried crop biomass capacity (level 1). A comprehensive assessment of the effect of all four system levels on the agroecosystem showed 2–35% higher effectiveness of the ternary crop.

## Figures and Tables

**Table 1:** Average air temperature (°C) and precipitation rate (mm) during multi-crop vegetative periods at Kaunas Meteorological Station.

Month/Year	2020	2021	2022	Long-Term Average
Air temperature (°C)				
March	3.3	1.6	1.5	2.1
April	6.9	6.2	6.2	6.4
May	10.5	11.4	11.0	11.0
June	19.0	19.5	17.7	18.7
July	17.4	22.6	17.9	19.3
August	18.7	16.5	20.9	18.7
September	14.8	11.4	10.6	12.3
Precipitation rate (mm)				
March	21.0	1.9	49.3	24.1
April	4.0	33.7	38.4	25.4
May	94.4	121.6	84.0	100.0
June	99.3	40.3	77.6	72.4
July	60.5	48.4	100.5	69.8
August	92.8	122.2	38.7	84.6
September	30.0	48.0	44.0	40.7

**Table 2:** Experimental treatments.

Biodiversity Level	Crops	Abbreviation and Number of Treatments
Single crop	Maize, hemp, faba bean (single crops)	M (1), H (2), FB (3)
Binary crop	Maize + hemp	M + H (4)
	Maize + faba bean	M + FB (5)
	Hemp + faba bean	H + FB (6)
Ternary crop	Maize + hemp + faba bean	M + H + FB (7)

**Table 3:** Tested variables and their variation.

Index	Variation	Unit	Index	Variation	Unit
Total dried crop biomass (end of vegetative season)	88.7–4401.6	g m^−2^	CO_2_ e-flux rate (middle of vegetative season)	2.4–4.68	µmol m^−2^ s^−1^
PAR at soil surface	2.3–27.1	%	Change in total nitrogen	−0.033–0.022	mg kg^−1^
Total crop density (end of vegetative season)	19–95	units m^−2^	Change in mobile phosphorus	−29.0–35.0	mg kg^−1^
Average crop height (end of vegetative season)	63.4–239.5	cm	Change in mobile potassium	−27.0–17.5	mg kg^−1^
Average leaf chlorophyll index (second half of crop vegetative season)	7.4–75.7	-	Change in mobile magnesium	−32.0–545	mg kg^−1^
Average leaf assimilative area (middle of vegetative season)	66.15–427.1	cm^−2^	Dried biomass of total weeds (end of vegetative season)	7.5–224.4	g m^−2^
Soil megastructure content (beginning of vegetative season)	31.9–52.6	%	Total number of weeds (end of vegetative season)	111.1–472.2	units m^−2^
Soil macrostructure content (beginning of vegetative season)	46.7–65.3	%	Average daily air temperature	1.5–22.6	°C
Soil microstructure content (beginning of vegetative season)	0.7–3.1	%	Precipitation rate	1.9–122.2	mm
Soil aggregate stability (beginning of vegetative season)	30.2–45.4	%	-	-	-

## Data Availability

Data is contained within the article.
